# Variation in Singlet Oxygen Generation in Dye-Conjugated Satellite Silver Nanoparticles on Silica Nanoparticles

**DOI:** 10.3390/ijms27167154

**Published:** 2026-08-10

**Authors:** Zahoor Muhammad, Akira Shinohara, Hideyuki Shinmori

**Affiliations:** 1 Department of Biotechnology, Faculty of Life and Environmental Science, Graduate Faculty of Interdisciplinary Research, University of Yamanashi, 4-4-37 Takeda, Kofu 400-8510, Yamanashi, Japan; 2 Sagami Chemical Research Institute, 2743-1 Hayakawa, Ayase 252-1193, Kanagawa, Japan

**Keywords:** silver nanoparticles, photosensitisers, singlet oxygen generation, plasmonic interactions, dye–metal distance, photodynamic nanomaterials

## Abstract

Understanding singlet oxygen (^1^O_2_) generation in plasmon-coupled photosensitiser systems is important for the design of photodynamic nanomaterials. In this study, silica nanoparticles decorated with satellite silver nanoparticle–photosensitiser conjugates (SiO_2_–Ag–dye) were prepared using three representative photosensitisers: Rose Bengal (RB), protoporphyrin IX (PpIX), and coumarin-3-carboxylic acid (C3CA). ^1^O_2_ generation measurements showed lower singlet oxygen quantum yields (***Φ*_Δ_**) for all nanoparticle-bound dyes than for the corresponding free dyes. Among the investigated systems, SiO_2_–Ag–PpIX retained approximately 80% of the ^1^O_2_ generation activity observed for the corresponding free dye (***Φ*_Δ_** = 0.45 ± 0.04), whereas SiO_2_–Ag–RB (***Φ*_Δ_** = 0.31 ± 0.15) and SiO_2_–Ag–C3CA (***Φ*_Δ_** = 0.12 ± 0.02) retained only approximately 40% of the corresponding free-dye activity. These results suggest that the photophysical behaviour and ^1^O_2_ generation efficiency of Ag-based dye–nanoparticle systems depend strongly on the immobilised photosensitiser. The findings provide insight into the interactions between plasmonic nanostructures and photosensitisers and may assist the future design of photodynamic nanomaterials. The present study should be regarded as a proof-of-concept demonstration of dye-dependent plasmonic modulation of ^1^O_2_ generation. Evaluation of practical photodynamic performance, photostability, and reusability will be the subject of future studies.

## 1. Introduction

Plasmonic metal nanoparticles have attracted significant attention due to their unique ability to capture and manipulate light at the nanometre scale [1,2]. When light interacts with the surface of metallic nanoparticles, the collective oscillation of conduction band electrons gives rise to localised surface plasmon resonance (LSPR) [3,4,5]. Among metals, silver is widely used because of its strong plasmonic response in the visible spectral region as well as its relatively low cost [6,7,8].

Singlet oxygen (^1^O_2_) is generated through energy transfer from a photoexcited photosensitiser to molecular oxygen and is widely used in photodynamic applications [9,10,11,12]. Because ^1^O_2_ generation strongly depends on excited-state relaxation processes, plasmonic nanoparticles have attracted attention as modulators of photodynamic activity through LSPR [13,14,15]. Upon light irradiation, the resulting localised electromagnetic fields can alter the excitation and relaxation behaviour of nearby photosensitisers depending on photosensitiser–metal separation distance [16,17].

Most previous studies have focused on controlling the dye–metal separation distance by varying the length of molecular linkers [18] or the thickness of dielectric shells surrounding metal nanoparticles [19], thereby balancing electromagnetic enhancement and non-radiative quenching. More recently, engineering plasmonic nanoarchitectures that promote interparticle plasmon coupling has emerged as an alternative strategy for generating highly enhanced electromagnetic fields, known as plasmonic hot spots, which are expected to further enhance the excitation efficiency of nearby photosensitisers. However, to the best of our knowledge, different photosensitisers don’t exist within the same hotspot-generating plasmonic platform.

Here, we investigate photosensitiser-dependent plasmonic effects using silica-supported satellite silver nanostructures functionalised with three representative photosensitising dyes: Rose Bengal (RB), protoporphyrin IX (PpIX), and coumarin-3-carboxylic acid (C3CA). The satellite Ag architecture was designed to exploit interparticle plasmon coupling while maintaining a common nanoparticle platform, enabling direct comparison of dye-dependent ^1^O_2_ generation under essentially identical plasmonic environments. This approach provides insight into the role of photosensitiser identity in determining plasmon-modulated photophysical behaviour and ^1^O_2_ generation.

## 2. Results

**Scheme 1:**
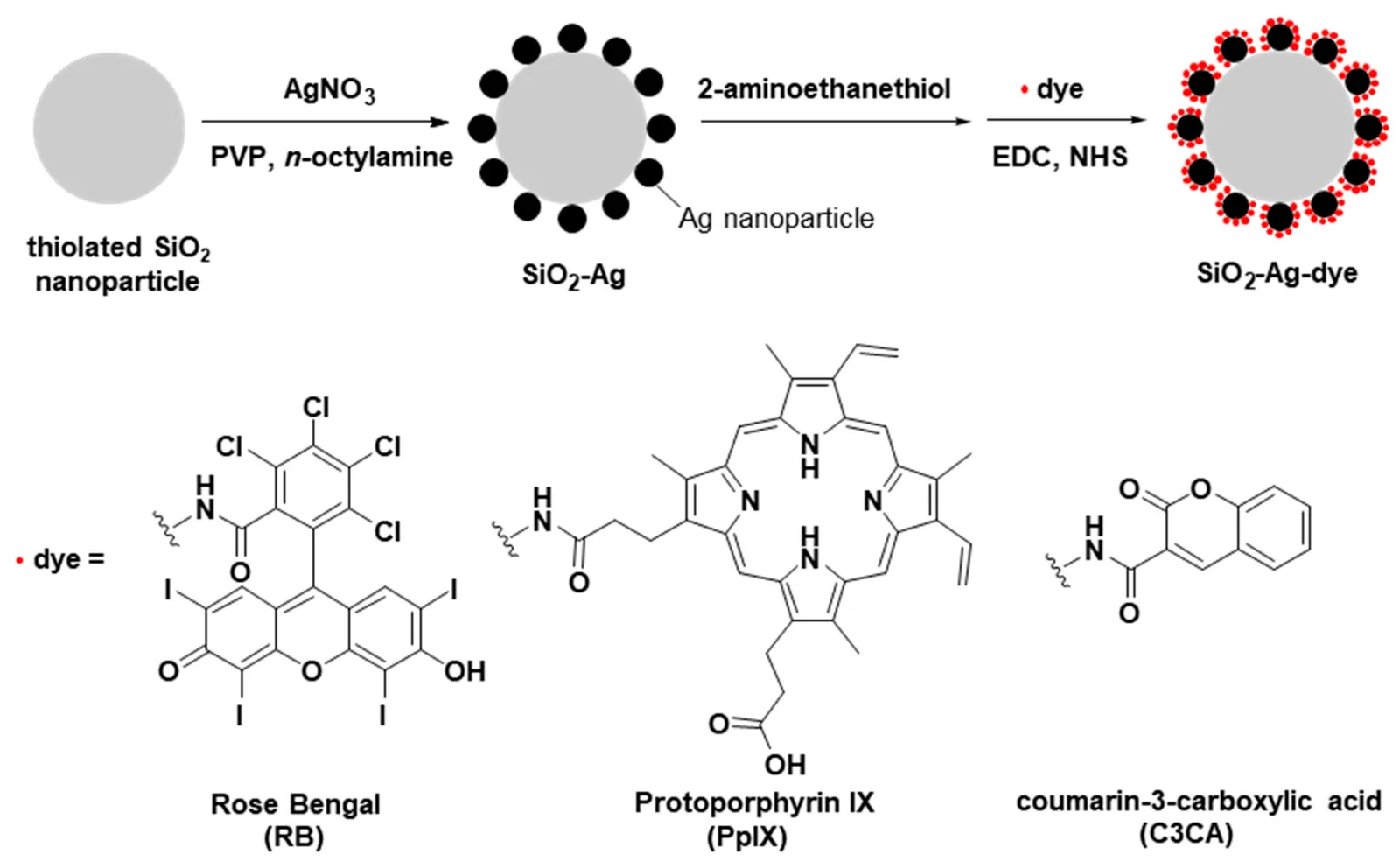
Synthesis of dye-conjugated satellite silver nanoparticles on silica nanoparticles (SiO_2_–Ag–dye).

**Figure 1:**
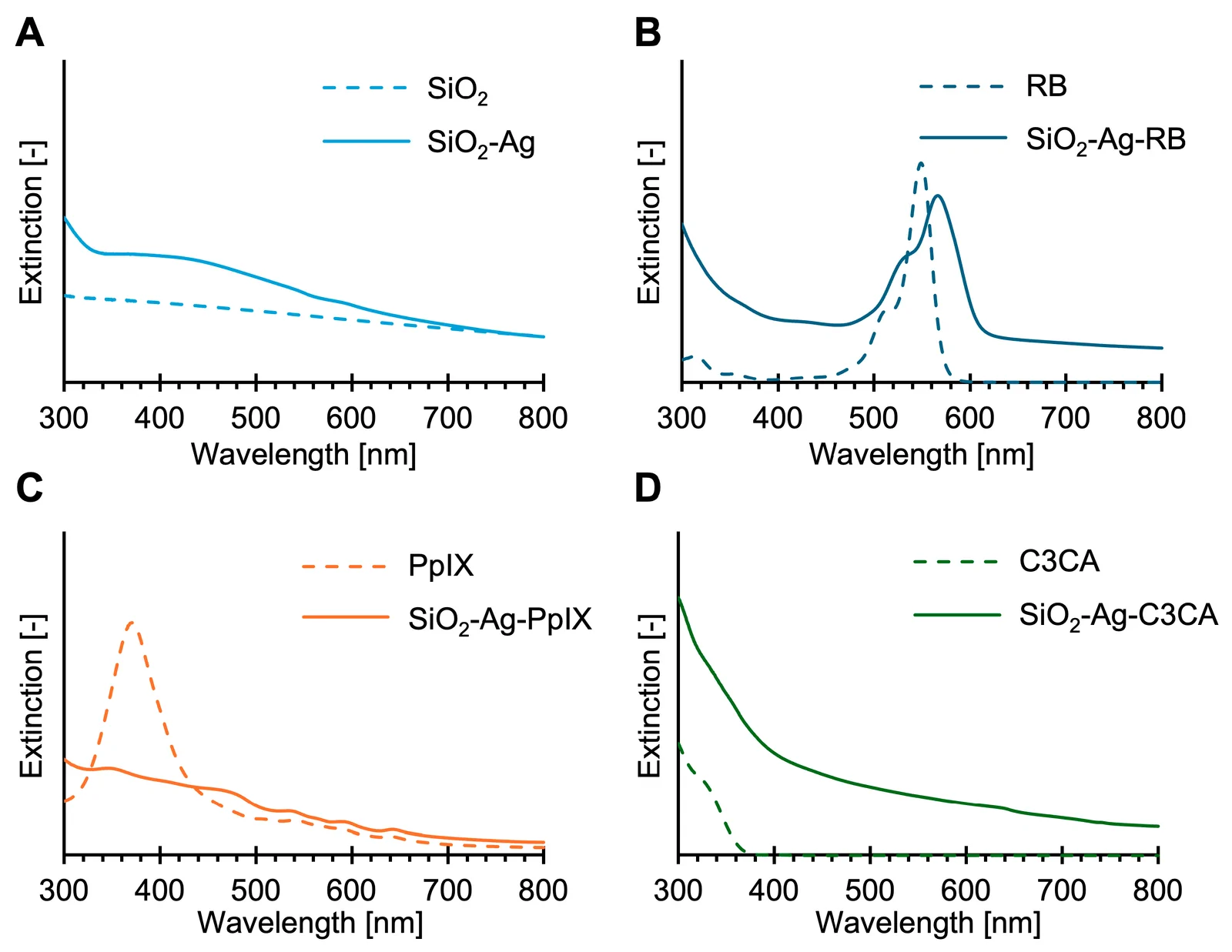
UV–vis extinction spectra of (**A**) SiO_2_ nanoparticles and SiO_2_–Ag, (**B**) free RB and SiO_2_–Ag–RB, (**C**) free PpIX and SiO_2_–Ag–PpIX, and (**D**) free C3CA and SiO_2_–Ag–C3CA.

Dye-conjugated satellite silver nanoparticles on silica nanoparticles (SiO_2_–Ag–dye) were prepared by deposition of silver nanoparticles onto thiolated silica nanoparticles [20] followed by surface amination [21] and covalent immobilisation [22] of the respective dyes (Scheme 1). The UV–vis extinction spectrum of bare SiO_2_ nanoparticles exhibited a featureless, monotonically decreasing profile toward longer wavelengths (Figure 1A), which is mainly attributable to Rayleigh scattering [23]. After deposition of the Ag satellite nanoparticles, a distinct plasmonic absorption band appeared at around 400 nm, indicating the formation of SiO_2_–Ag nanoparticles. The UV–vis spectrum of SiO_2_–Ag–RB showed the characteristic absorption band of RB, with a slight red shift and broadening compared with that of free RB (Figure 1B). In contrast, the Soret band of PpIX around 380 nm was markedly suppressed in the SiO_2_–Ag–PpIX spectrum (Figure 1C), which is consistent with aggregation of PpIX [24]. The SiO_2_–Ag–C3CA sample showed the characteristic absorption of C3CA without a pronounced spectral change compared with the free dye (Figure 1D).

### 2.1. Morphological and Elemental Characterisation of SiO_2_–Ag–Dye Nanoconjugates

**Figure 2:**
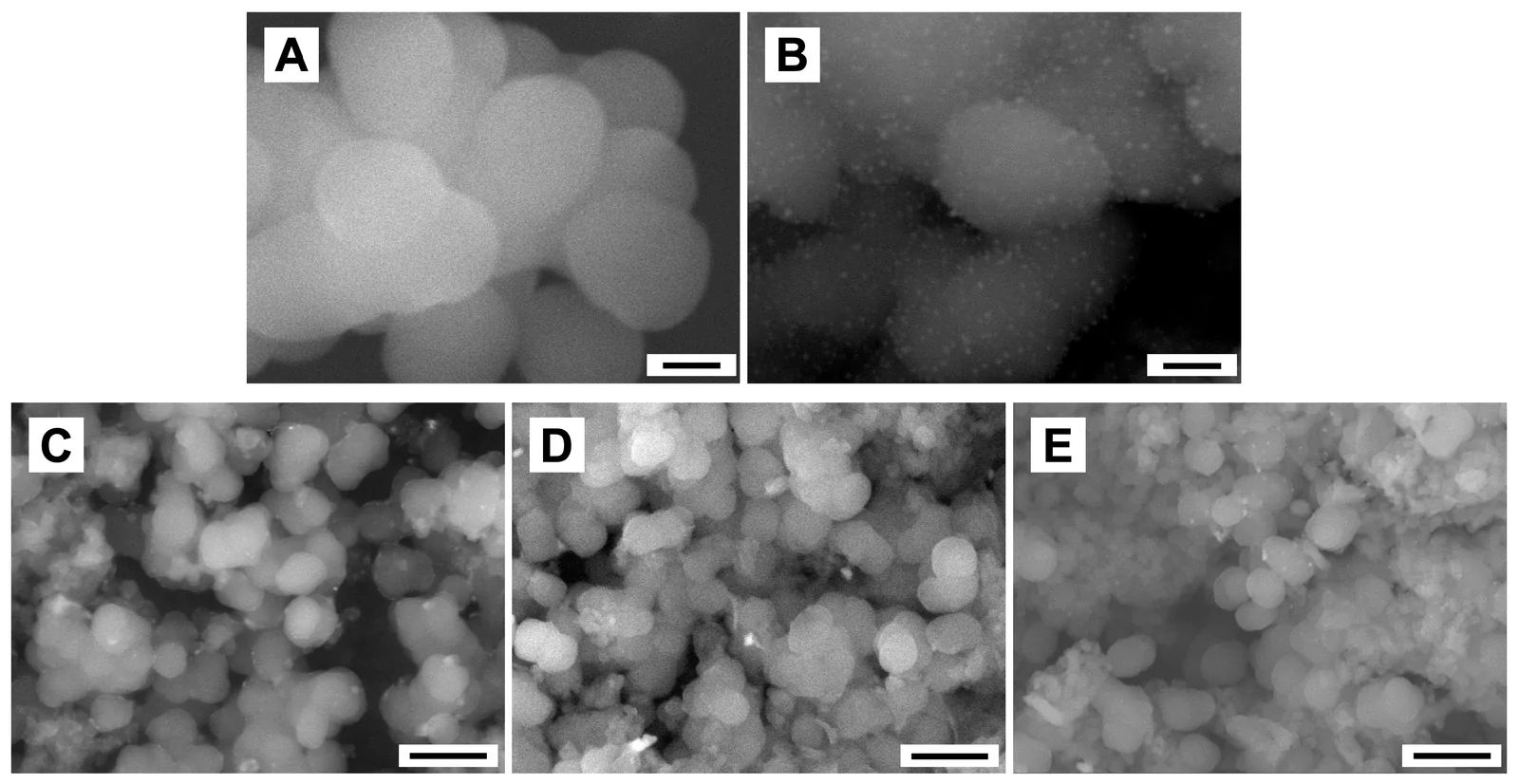
SEM images of (**A**) thiolated SiO_2_ nanoparticles, (**B**) SiO_2_–Ag, (**C**) SiO_2_–Ag–RB, (**D**) SiO_2_–Ag–PpIX, and (**E**) SiO_2_–Ag–C3CA. Satellite silver nanoparticles are observed on the SiO_2_ surface after Ag deposition, while the overall particle morphology is largely retained after dye immobilisation. Scale bars: (**A**,**B**) 100 nm; (**C**–**E**) 500 nm.

The morphology of SiO_2_–Ag was examined by scanning electron microscopy (SEM). Before Ag deposition, the SiO_2_ nanoparticles exhibited a uniform spherical morphology with smooth surfaces (Figure 2A). The average diameter was 206 ± 23 nm, as determined from the size distribution histogram (Figure S1 in the Supporting Information). After Ag deposition, the overall spherical morphology was retained, while satellite Ag nanoparticles with an average diameter of 5.6 ± 1.7 nm were observed on the surface (Figure 2B and Figure S2 in the Supporting Information). No apparent morphological change was observed after dye immobilisation (Figure 2C–E).

Dynamic light scattering (DLS) measurements (n = 3) showed that the average hydrodynamic diameter increased from 217 ± 5 nm for thiolated SiO_2_ nanoparticles to 279 ± 93 nm for SiO_2_–Ag, and further to 317 ± 21, 343 ± 57, and 330 ± 11 nm for SiO_2_–Ag–RB, SiO_2_–Ag–PpIX, and SiO_2_–Ag–C3CA, respectively (Figures S3–S7 in the Supporting Information), whereas SEM observations revealed no apparent change in particle morphology. This discrepancy is likely attributable to the hydrodynamic nature of DLS measurements, reflecting slight aggregation and/or changes in the surface solvation layer rather than an increase in the physical particle size.

Zeta potential measurements (n = 3) showed systematic changes following Ag deposition and dye immobilisation, changing from −9.6 ± 2.0 mV for thiolated SiO_2_ nanoparticles to −5.1 ± 5.5 mV for SiO_2_–Ag, −3.0 ± 0.9 mV for SiO_2_–Ag–RB, +0.2 ± 6.8 mV for SiO_2_–Ag–PpIX, and +12.8 ± 2.3 mV for SiO_2_–Ag–C3CA, indicating changes in the surface chemical environment. In particular, the surface charge became progressively less negative after Ag deposition and varied depending on the immobilised photosensitiser, suggesting successful surface modification.

The relatively small absolute zeta potential values (approximately −10 to +13 mV) also suggest limited electrostatic stabilisation [25]. Therefore, factors other than electrostatic stabilisation, such as steric effects arising from surface-bound molecules, are likely to contribute to the colloidal stability, while slight aggregation under the measurement conditions cannot be excluded. This interpretation is consistent with the increased hydrodynamic diameters observed by DLS (Figures S5–S7 in the Supporting Information).

EDX analysis was performed to confirm the elemental composition (Figures S8–S12 in the Supporting Information). No Ag signal was detected for bare SiO_2_. Ag signals were observed after Ag deposition and remained detectable after dye immobilisation, confirming the retention of Ag nanoparticles on the SiO_2_ surface throughout the immobilisation process. Semi-quantitative EDX analysis for SiO_2_–Ag is summarised in Table S1 of the Supporting Information. Based on the Ag/Si atomic ratio, the number of Ag satellite nanoparticles was semi-quantitatively estimated to be approximately 460 per SiO_2_ core. However, the crystalline structure of the Ag satellite nanoparticles was not directly characterised by X-ray diffraction (XRD), selected-area electron diffraction (SAED), or high-resolution transmission electron microscopy (HRTEM).

### 2.2. FTIR

**Figure 3:**
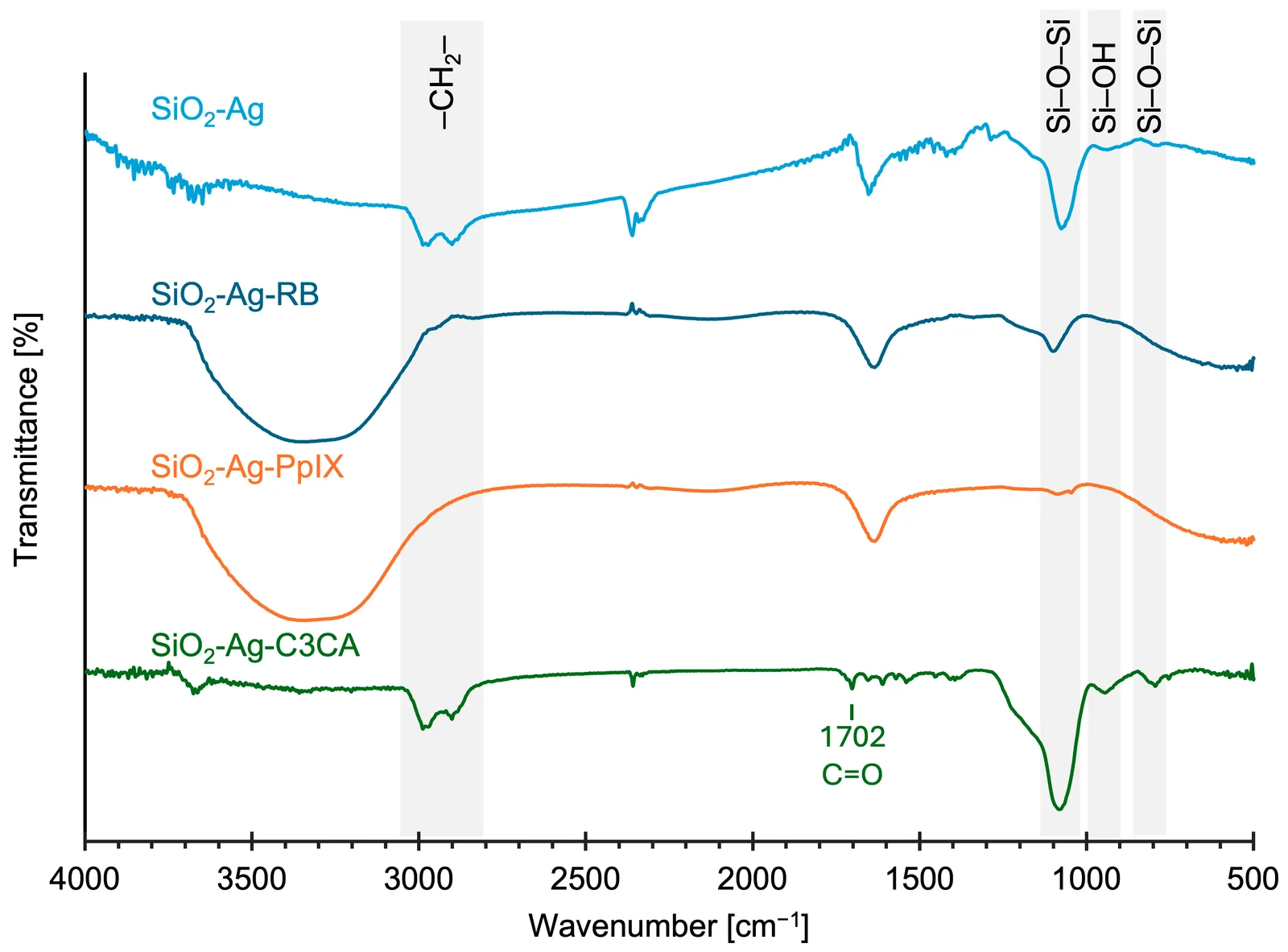
FTIR spectra of SiO_2_–Ag and SiO_2_–Ag–dyes. Spectra are vertically offset for clarity.

To evaluate the dye immobilisation on the SiO_2_–Ag, FTIR spectra were recorded (Figure 3). For comparison, the FTIR spectra of the free dyes are shown in Figure S13 in the Supporting Information. Compared with bare SiO_2_–Ag, SiO_2_–Ag–C3CA exhibited additional absorption bands in the 1700–1000 cm^−1^ region that were attributable to immobilised C3CA. In contrast, no obvious spectral changes associated with dye immobilisation were observed for SiO_2_–Ag–RB or SiO_2_–Ag–PpIX.

This difference is likely due to the lower dye loading of RB and PpIX on the SiO_2_–Ag compared with C3CA. Indeed, considering the molar extinction coefficients of the dyes (RB: 9.0 × 10^4^ L/mol·cm at 560 nm in basic ethanol [26]; PpIX: approximately 1.0 × 10^4^ L/mol·cm for the Q bands, in water [27]; C3CA: approximately 1.0 × 10^4^ L/mol·cm at 300 nm in phosphate buffer [28]), the absorption intensities shown in Figure 1 are consistent with lower dye loadings for SiO_2_–Ag–RB and SiO_2_–Ag–PpIX than for SiO_2_–Ag–C3CA. It should be noted, however, that the absorption spectra of nanoparticle dispersions are also influenced by light scattering and possible dye aggregation. Therefore, this comparison should be regarded as a semi-quantitative indication of the relative dye loading.

### 2.3. Fluorescence Spectra

**Figure 4:**
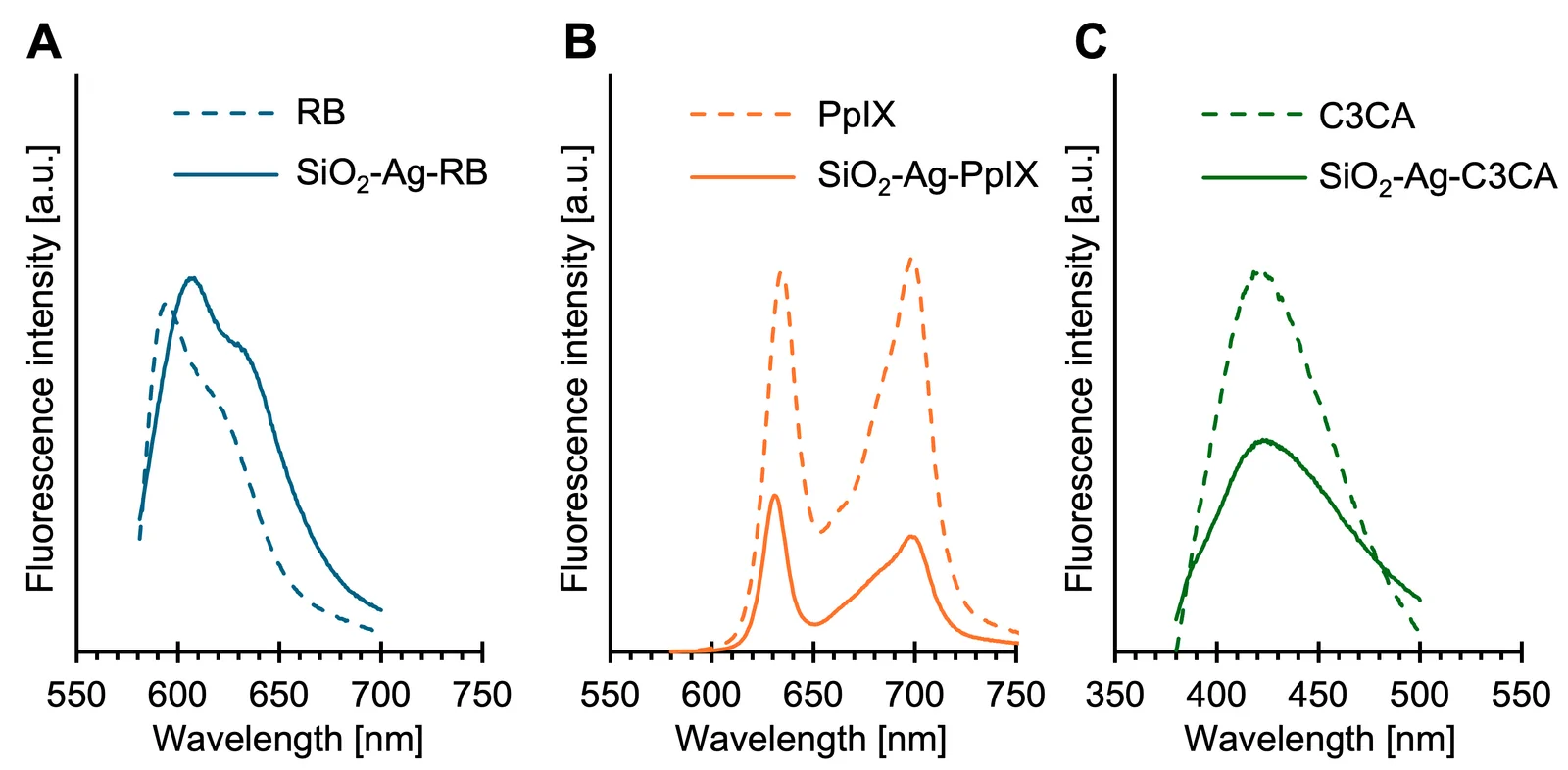
Fluorescence emission spectra of free dyes and corresponding SiO_2_–Ag–dye nanoparticles. Excitation wavelengths were 560 nm for RB (**A**), 385 nm for PpIX (**B**), and 360 nm for C3CA (**C**), respectively. Emission intensities were normalised to the absorbance at the excitation wavelength.

Fluorescence spectroscopy was used to evaluate the photophysical changes after dye immobilisation. SiO_2_–Ag–RB exhibited a broader emission profile with only a modest change in fluorescence intensity compared with free RB (Figure 4A). In contrast, SiO_2_–Ag–PpIX showed strong suppression of both emission bands (Figure 4B). SiO_2_–Ag–C3CA showed a slight red shift in the emission maximum together with a decrease in fluorescence intensity (Figure 4C). These observations indicate that the influence of the Ag nanostructures on the excited-state behaviour depends strongly on the immobilised dye.

### 2.4. ^1^O_2_ Generation

**Figure 5:**
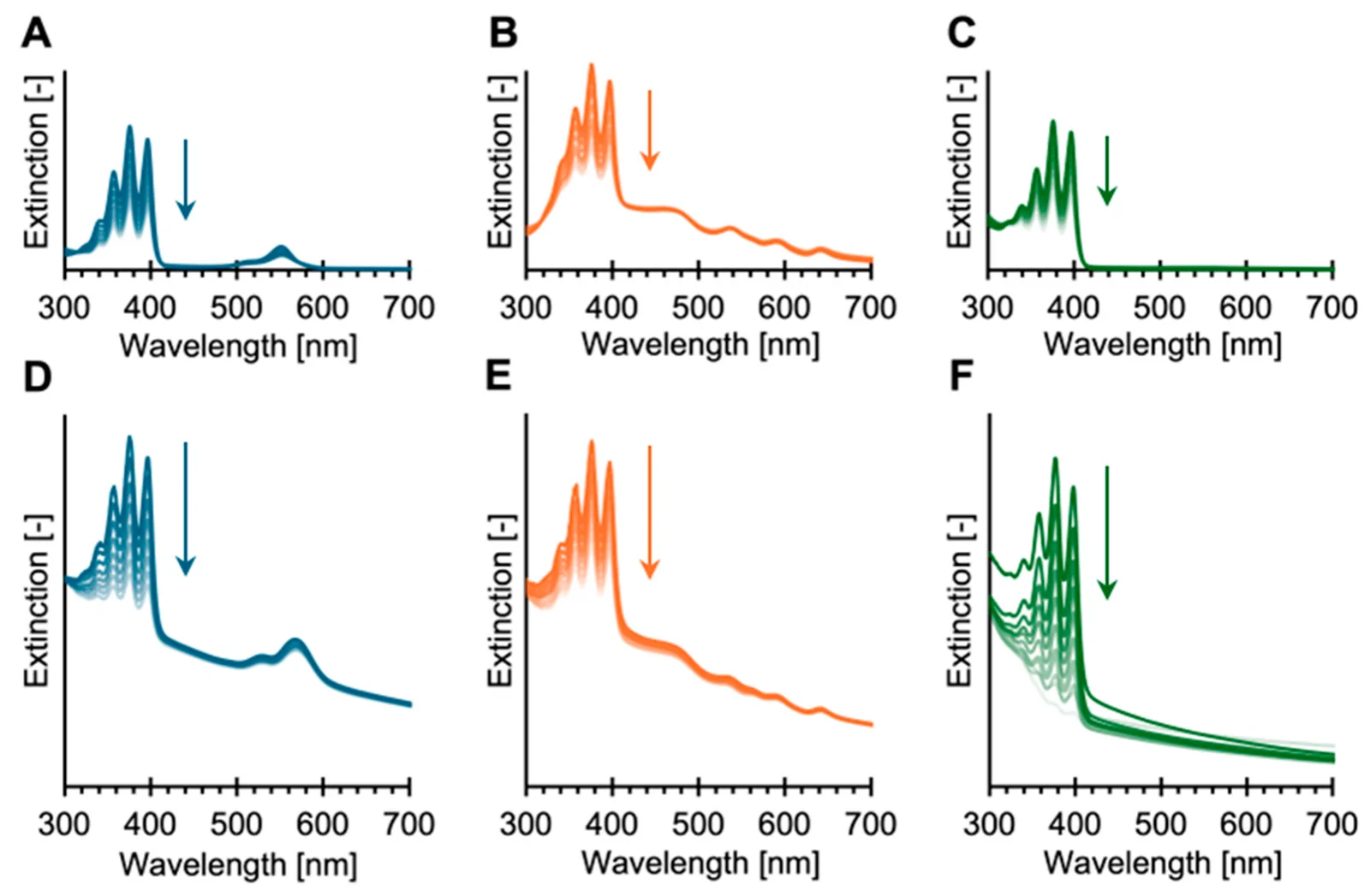
Bleaching of 9,10-anthracenediylbis(methylene)dimalonic acid (ABDA) during photosensitised ^1^O_2_ generation in the presence of (**A**) RB, (**B**) PpIX, (**C**) C3CA, (**D**) SiO_2_–Ag–RB, (**E**) SiO_2_–Ag–PpIX, and (**F**) SiO_2_–Ag–C3CA. UV–vis extinction spectra were recorded at 1 min intervals.

^1^O_2_ generation by SiO_2_–Ag–dye was evaluated using 9,10-anthracenediylbis(methylene)dimalonic acid (ABDA) as a chemical probe (Figure 5). The decrease in the ABDA absorption band during photoirradiation was used to assess ^1^O_2_ generation, and singlet oxygen quantum yields (***Φ*_Δ_**) were estimated from the corresponding degradation kinetics (Table 1). A gradual decrease in ABDA absorbance was observed for all samples, consistent with ^1^O_2_ generation. The estimated ***Φ*_Δ_** values depended strongly on the immobilised photosensitiser, with SiO_2_–Ag–PpIX exhibiting the highest activity, whereas SiO_2_–Ag–RB and SiO_2_–Ag–C3CA showed lower values. These results suggest that the efficiency of ^1^O_2_ generation in the SiO_2_–Ag–dye nanoparticles is strongly influenced by the identity of the immobilised dye.

## 3. Discussion

The fluorescence and ^1^O_2_ generation properties of the SiO_2_–Ag–dye depended strongly on the immobilised dye. Fluorescence measurements revealed dye-dependent changes in emission intensity and spectral shape after immobilisation on the Ag nanostructures. Similarly, the estimated ***Φ*_Δ_** values differed among the three systems, with SiO_2_–Ag–PpIX exhibiting higher activity than SiO_2_–Ag–RB and SiO_2_–Ag–C3CA.

These observations indicate that the observed photophysical behaviour cannot be attributed solely to the plasmonic Ag nanostructure but is also strongly influenced by the intrinsic photophysical properties of the immobilised dye. Previous studies have shown that plasmonic nanostructures can either enhance or suppress fluorescence and ^1^O_2_ generation depending on factors such as the dye–metal separation distance [32], spectral overlap [33], molecular orientation [34], and excited-state dynamics [35]. In the present study, all dyes were immobilised on the same SiO_2_–Ag platform under comparable preparation conditions, yet markedly different photophysical behaviours were observed. This suggests that the intrinsic photophysical properties of the photosensitisers, together with their interaction with the Ag surface, are likely to play an important role in determining the balance between radiative emission, non-radiative energy dissipation, and ^1^O_2_ generation.

Another limitation of the present study is that the crystalline structure of the Ag satellite nanoparticles was not directly confirmed because XRD, SAED, and HRTEM measurements were not performed. Further studies involving controlled dye–metal separation distances, surface architectures, and time-resolved fluorescence measurements to determine excited-state lifetimes will be important for clarifying the mechanism underlying the observed photophysical behaviour and ^1^O_2_ generation in plasmonic dye–nanoparticle systems.

## 4. Materials and Methods

### 4.1. Chemicals and Reagents

Tetraethyl orthosilicate (TEOS, Kishida Chemical, Osaka, Japan, >99.0%), 3-mercaptopropyltrimethoxysilane (TCI, Tokyo, Japan, >96.0%), poly(vinylpyrrolidone) (PVP, K30, TCI, Tokyo, Japan, average molecular weight ~40,000), silver nitrate (TCI, Tokyo, Japan, >99.8%), *n*-octylamine (TCI, Tokyo, Japan, >98.0%), 2-aminoethanethiol (TCI, Tokyo, Japa, >95.0%), Rose Bengal (RB, Nacalai Tesque, Kyoto, Japan, >85.0%), 1-(3-dimethylaminopropyl)-3-ethylcarbodiimide hydrochloride (EDC·HCl, TCI, Tokyo, Japan, >98.0%), *N*-hydroxysuccinimide (NHS, Wako, Osaka, Japan, >98.0%), protoporphyrin IX disodium salt (PpIX, TCI, Tokyo, Japan, >95.0%), coumarin-3-carboxylic acid (C3CA, TCI, Tokyo, Japan, >98.0%), and 9,10-anthracenediylbis(methylene)dimalonic acid (ABDA, Santa Cruz, Dallas, TX, USA, >90%) were used as received. All other reagents and solvents were purchased from commercial suppliers and used without further purification unless otherwise stated.

### 4.2. Preparation of Dye-Conjugated Samples

#### 4.2.1. Thiolated SiO_2_ Nanoparticles [20]

A reaction mixture containing tetraethyl orthosilicate (TEOS, 1.6 mL, 7.2 mmol), ethanol (40 mL), and 30% aqueous ammonium hydroxide (3 mL, 44 mmol) was stirred at room temperature for 16 h. The resulting SiO_2_ nanoparticles were collected by centrifugation (4500× *g*, 30 min) and washed with ethanol (3 × 50 mL). The purified SiO_2_ nanoparticles were redispersed in ethanol (10 mL), followed by the addition of 3-mercaptopropyltrimethoxysilane (MPTS, 300 μL, 1.6 mmol). The mixture was stirred overnight at room temperature. The resulting thiolated SiO_2_ nanoparticles were collected by centrifugation (4500× *g*, 30 min) and washed with ethanol (3 × 50 mL). The purified nanoparticles were dried under reduced pressure and stored at 4 °C until use. The average diameter of the SiO_2_ nanoparticles was 206 ± 23 nm (N = 21), as determined by SEM (Figure S1 in the Supporting Information).

#### 4.2.2. Deposition of Ag [20]

The thiolated SiO_2_ nanoparticles (10 mg) were dispersed in ethylene glycol (25 mL) containing poly(vinylpyrrolidone) (PVP, 10 mg). In a separate vessel, silver nitrate (30 mg, 0.18 mmol) was dissolved in ethylene glycol (25 mL). The two solutions were combined and stirred at room temperature for 30 min, followed by addition of *n*-octylamine (41 μL, 0.25 mmol). The reaction mixture gradually changed from brown to green, indicating formation of Ag nanoparticles on the SiO_2_ surface. The resulting SiO_2_–Ag was collected by centrifugation (10,000× *g*, 1 h), washed with ethanol (2 × 50 mL), and redispersed in ethanol (10 mL). The average diameter of the satellite Ag nanoparticles was 5.6 ± 1.7 nm (N = 190), as determined by SEM (Figure S2 in the Supporting Information).

#### 4.2.3. Dye Loading

Prior to dye immobilisation, SiO_2_–Ag nanoparticles were functionalised with amino groups [22]. 2-Aminoethanethiol (12 mg, 0.148 mmol) was added to the SiO_2_–Ag dispersion (10 mL), and the mixture was stirred overnight at room temperature. The resulting aminated SiO_2_–Ag was repeatedly washed with ethanol. After purification, the aminated SiO_2_–Ag was redispersed in ethanol/water (1:1, *v*/*v*, 6 mL) and stored at 4 °C prior to use.

RB, PpIX, and C3CA were immobilised onto aminated SiO_2_–Ag nanoparticles through EDC/NHS-mediated coupling reactions [29]. For preparation of SiO_2_–Ag–RB, RB (0.66 mg, 0.65 μmol) in water (6 mL) was activated by addition of EDC·HCl (12 mg, 0.063 mmol) and NHS (7 mg, 0.061 mmol) under stirring for 30 min at room temperature. The activated dye solution was then added dropwise to the aminated SiO_2_–Ag dispersion prepared above (6 mL) and stirred overnight at 4 °C. The resulting SiO_2_–Ag–RB was collected by centrifugation (4500× *g*, 30 min) and washed with ethanol (3 × 15 mL). After purification, the SiO_2_–Ag–RB was redispersed in water (6 mL) and stored at 4 °C prior to use.

For preparation of SiO_2_–Ag–PpIX, protoporphyrin IX disodium salt (0.60 mg, 0.99 μmol) in acetonitrile/water (30:70, *v*/*v*, 6 mL) was activated by addition of EDC·HCl (12 mg, 0.063 mmol) and NHS (7 mg, 0.061 mmol) under stirring for 30 min at room temperature. The activated dye solution was then added dropwise to the aminated SiO_2_–Ag nanoparticle dispersion prepared above (6 mL) and stirred overnight at 4 °C in the dark. The resulting SiO_2_–Ag–PpIX was collected by centrifugation (4500× *g*, 30 min) and washed with water (3 × 15 mL). After purification, the SiO_2_–Ag–PpIX was redispersed in acetonitrile/water (30:70, *v*/*v*, 6 mL) and stored at 4 °C prior to use.

For preparation of SiO_2_–Ag–C3CA, C3CA (19 mg, 0.10 mmol) in acetonitrile/water (30:70, *v*/*v*, 10 mL) was activated by addition of EDC·HCl (12 mg, 0.063 mmol) and NHS (7 mg, 0.061 mmol) under stirring for 30 min at room temperature. The aminated SiO_2_–Ag nanoparticle dispersion prepared above (6 mL) was centrifuged (4500× *g*, 30 min) and redispersed in acetonitrile/water (30:70, *v*/*v*, 10 mL). The activated dye solution was then added dropwise to this dispersion under stirring, and the mixture was stirred overnight at room temperature in the dark. The resulting SiO_2_–Ag–C3CA was collected by centrifugation (4500× *g*, 30 min) and washed with water (1 × 15 mL) and ethanol (2 × 15 mL). After purification, the SiO_2_–Ag–C3CA was redispersed in acetonitrile/water (30:70, *v*/*v*, 10 mL) and stored at 4 °C prior to use.

### 4.3. Spectroscopic Characterisation

UV–vis extinction spectra were recorded on a Shimadzu UV-1800 spectrophotometer (Shimadzu, Kyoto City, Japan) using quartz cuvettes with a path length of 1 cm. Fluorescence spectra were recorded on a Shimadzu RF-6000 fluorescence spectrophotometer (Shimadzu, Kyoto, Japan). Fourier-transform infrared (FTIR) spectra of the nanoparticles were recorded using a Jasco FT/IR-4100 spectrometer (Jasco, Tokyo, Japan). The nanoparticles were collected by centrifugation, and FTIR spectra of the resulting pellets were recorded in attenuated total reflectance (ATR) mode.

Approximate dye loadings were estimated from the background-subtracted UV–vis extinction using the molar extinction coefficients of the corresponding free dyes (RB: 9.0 × 10^4^ L/mol·cm at 560 nm in basic ethanol; PpIX: approximately 1.0 × 10^4^ L/mol·cm for the Q bands in water; C3CA: approximately 1.0 × 10^4^ L/mol·cm at 300 nm in phosphate buffer) [26,27,28]. The estimated dye loadings were 35.2, 25.1, and 67.9 μg/mg SiO_2_ for SiO_2_–Ag–RB, SiO_2_–Ag–PpIX, and SiO_2_–Ag–C3CA, respectively. These values were calculated using the Beer–Lambert law from nanoparticle dispersions normalised to 1 mg/mL and should be regarded as approximate because the extinction coefficients of the corresponding free dyes were used.

### 4.4. Morphological and Elemental Characterisation

SEM images were obtained using a JEOL JSM-IT700HR scanning electron microscope (JEOL, Tokyo, Japan). Elemental composition was determined using a Malvern Panalytical Epsilon1 spectrophotometer (Malvern Panalytical, Tokyo, Japan).

### 4.5. ^1^O_2_ Measurements

All ***Φ*_Δ_** measurements were performed in a dark room at a maintained temperature (25 °C). To the properly diluted (absorbance < 1) sample dispersion, ABDA solution was added to make its concentration 50 μmol/L. The sample was filled in a quartz cuvette (1 cm × 1 cm) and irradiated using a Horiba Optical ModuleX (Horiba, Kyoto, Japan) (Xe short arc lamp, 500 W). A 500 nm long-pass filter (Y-50 (AGC Techno Glass, Shizuoka, Japan)) was used for RB, SiO_2_–Ag–RB, PpIX, and SiO_2_–Ag–PpIX, whereas a 350 nm band-pass filter (UV-D35 (AGC Techno Glass, Shizuoka, Japan)) was used for C3CA and SiO_2_–Ag–C3CA. The spectral intensity distributions of the irradiation light after filtering, together with the transmission spectra of the optical filters, are shown in Figure S14 (in the Supporting Information). The light irradiation period was controlled by a shutter, and the decrease in ABDA absorbance at 378 nm was monitored by UV–vis spectroscopy. The ***Φ*_Δ_**of the SiO_2_–Ag–dye nanoconjugates was determined by a relative method using the corresponding free dyes as references [15].

## 5. Conclusions

In this study, dye-functionalised SiO_2_–Ag nanoparticles incorporating Rose Bengal (RB), protoporphyrin IX (PpIX), and coumarin-3-carboxylic acid (C3CA) were prepared, and their fluorescence and ^1^O_2_ generation properties were examined. The nanoparticles retained Ag satellite structures after dye immobilisation, and spectral measurements confirmed dye-dependent optical features. Fluorescence measurements showed dye-dependent changes in emission intensity and spectral shape after immobilisation. ABDA assays further revealed that the ***Φ*_Δ_** of the SiO_2_–Ag–dye nanoparticles varied depending on the immobilised dye, with SiO_2_–Ag–PpIX showing higher activity than SiO_2_–Ag–RB and SiO_2_–Ag–C3CA.

These results show that the photophysical behaviour and ^1^O_2_ generation efficiency of SiO_2_–Ag–dye nanoparticles are strongly influenced by photosensitiser identity. Further studies with controlled dye–metal separation distances and surface structures will be useful for clarifying the factors governing fluorescence and ^1^O_2_ generation in plasmonic photosensitiser systems.

The present work provides a proof-of-concept for investigating dye-dependent plasmonic modulation of ^1^O_2_ generation using satellite Ag nanostructures. Future studies will focus on evaluating practical photodynamic performance, photostability, and reusability. Nevertheless, because the ***Φ*_Δ_** values were obtained from two independent experiments (n = 2), and dye loading, Ag loading, and irradiance were not quantitatively determined, the present results should be regarded as semi-quantitative rather than fully optimised photodynamic performance data.

## Figures and Tables

**Table 1:** Singlet oxygen quantum yield (***Φ*_Δ_**) for free dyes and SiO_2_–Ag–dye.

Sample	*Φ*_Δ_	Reference
RB	0.75	[29]
PpIX	0.56	[30]
C3CA	0.28	[31]
SiO_2_–Ag–RB	0.31 ± 0.15	
SiO_2_–Ag–PpIX	0.45 ± 0.04	
SiO_2_–Ag–C3CA	0.12 ± 0.02	

The ***Φ*_Δ_** values of the samples were determined by the relative method based on ABDA photobleaching, using the corresponding free dye (RB, PpIX, or C3CA) as the reference. Data are presented as mean ± SD from two independent experiments (n = 2).

## Data Availability

The original contributions presented in this study are included in the article/Supplementary Material. Further inquiries can be directed to the corresponding author.

## Supplementary Materials

The following supporting information can be downloaded at https://www.mdpi.com/article/10.3390/ijms27167154/s1.
