# Supplementary material for: Variation in Singlet Oxygen Generation in Dye-Conjugated Satellite Silver Nanoparticles on Silica Nanoparticles
Source: Int J Mol Sci. 2026 Aug 10;27(16):7154. doi: 10.3390/ijms27167154 (PMC13513667; doi:10.3390/ijms27167154)
Supplement: Supplementary file 1 [file ijms-27-07154-s001.zip › ijms-4412954-supplementary.pdf]

Supporting Information

Variation in Singlet Oxygen Generation in Dye-Conjugated Satellite Silver Nanoparticles on Silica Nanoparticles

Muhammad Zahoor[1], Akira Shinohara[1,2], Hideyuki Shinmori[1,*]

[1] Department of Biotechnology, Faculty of Life and Environmental Science, Graduate Faculty of Interdisciplinary Research, University of Yamanashi, 4-4-37 Takeda, Kofu, Yamanashi 400-8510 Japan

[2] Sagami Chemical Research Institute, 2743-1 Hayakawa, Ayase, Kanagawa 252-1193 Japan

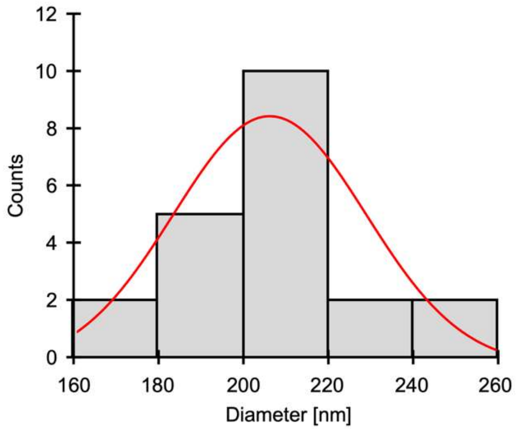

Figure S1. Particle size distribution of SiO_2 nanoparticles.

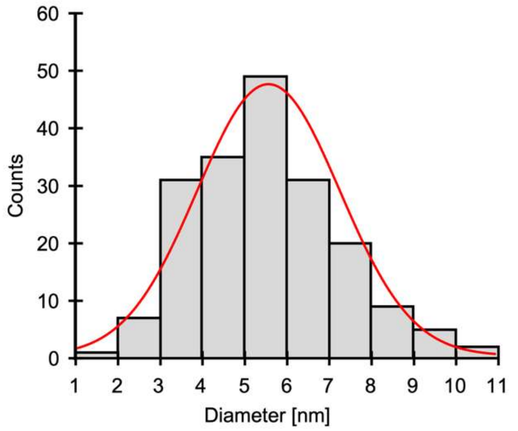

Figure S2. Particle size distribution of satellite Ag nanoparticles.

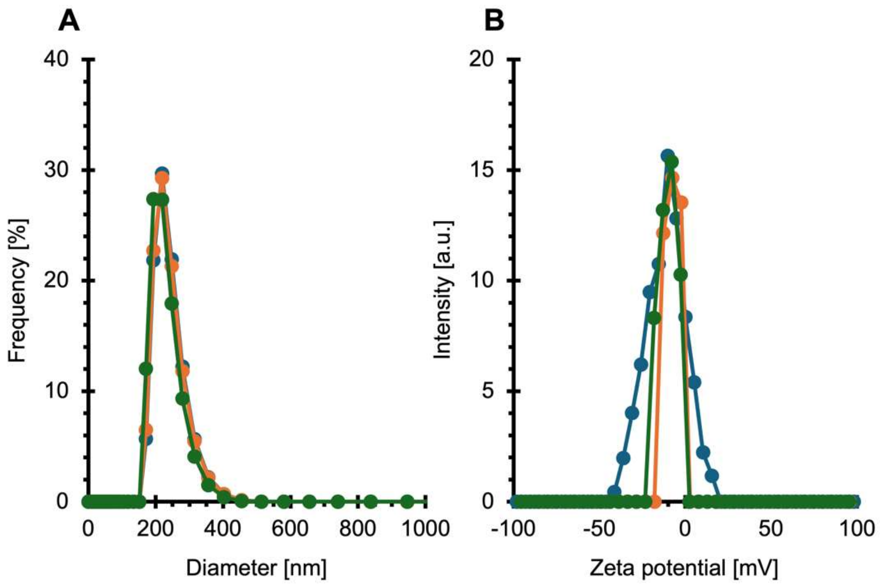

Figure S3. (A) Dynamic light scattering (DLS) particle size distributions and (B) zeta potential distributions of SiO_2 nanoparticles (after thiolation). The average hydrodynamic diameter and zeta potential, determined from three independent measurements, were 217 ± 5 nm and −9.6 ± 2.0 mV, respectively.

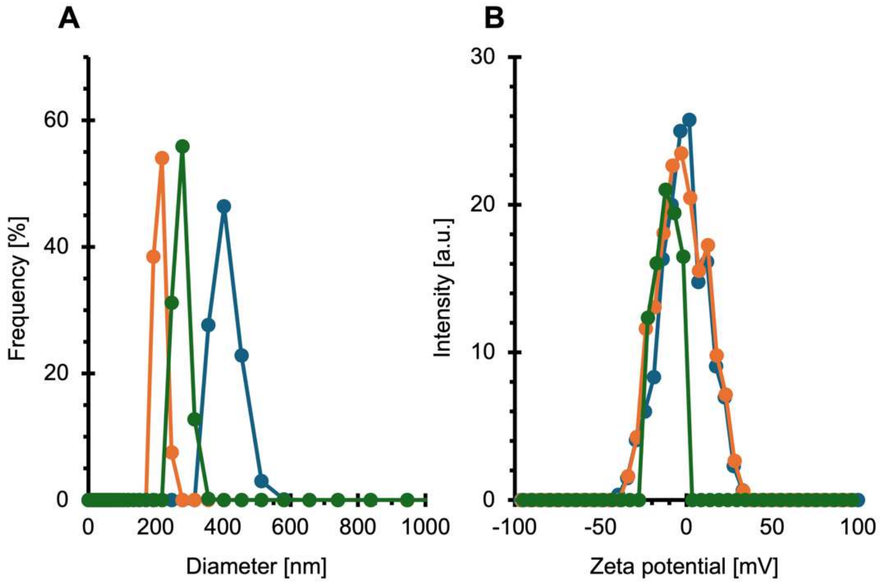

Figure S4. (A) Dynamic light scattering (DLS) particle size distributions and (B) zeta potential distributions of SiO_2–Ag. The average hydrodynamic diameter and zeta potential, determined from three independent measurements, were 279 ± 93 nm and −5.1 ± 5.5 mV, respectively.

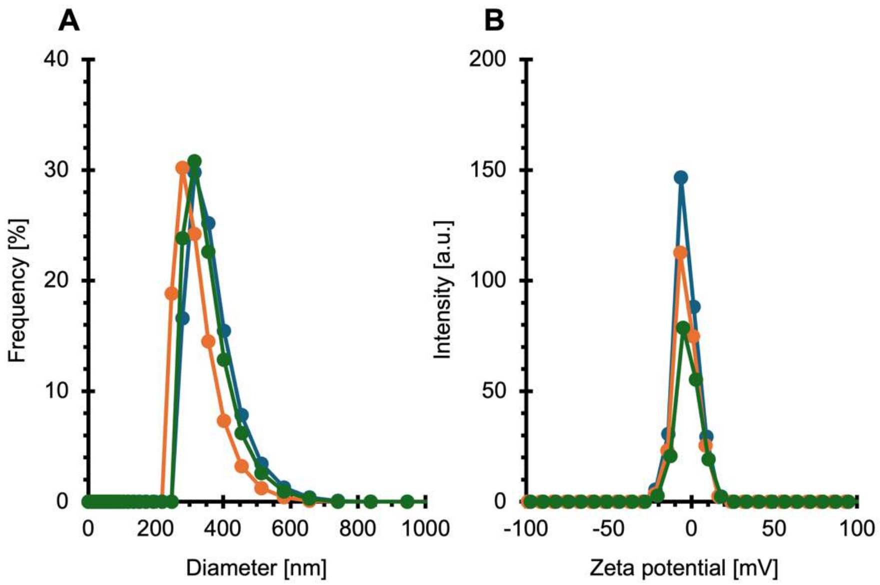

Figure S5. (A) Dynamic light scattering (DLS) particle size distributions and (B) zeta potential distributions of SiO_2–Ag–RB. The average hydrodynamic diameter and zeta potential, determined from three independent measurements, were 317 ± 21 nm and −3.0 ± 0.9 mV, respectively.

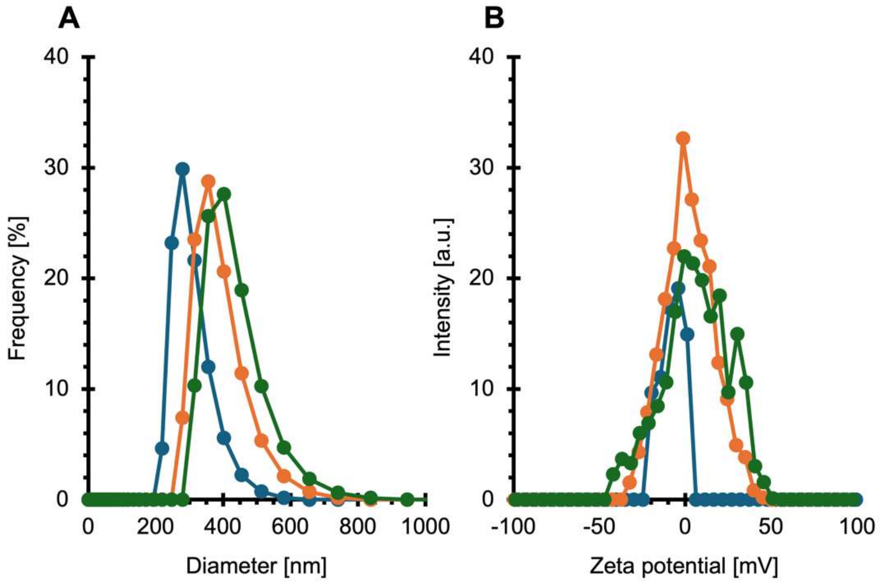

Figure S6. (A) Dynamic light scattering (DLS) particle size distributions and (B) zeta potential distributions of SiO_2–Ag–PpIX. The average hydrodynamic diameter and zeta potential, determined from three independent measurements, were 343 ± 57 nm and +0.2 ± 6.8 mV, respectively.

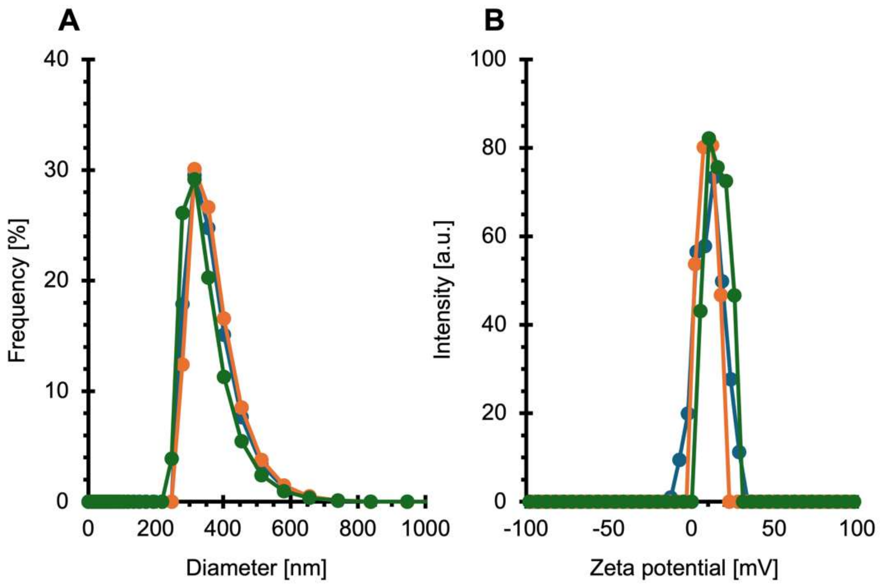

Figure S7. (A) Dynamic light scattering (DLS) particle size distributions and (B) zeta potential distributions of SiO_2–Ag–C3CA. The average hydrodynamic diameter and zeta potential, determined from three independent measurements, were 330 ± 11 nm and +12.8 ± 2.3 mV, respectively.

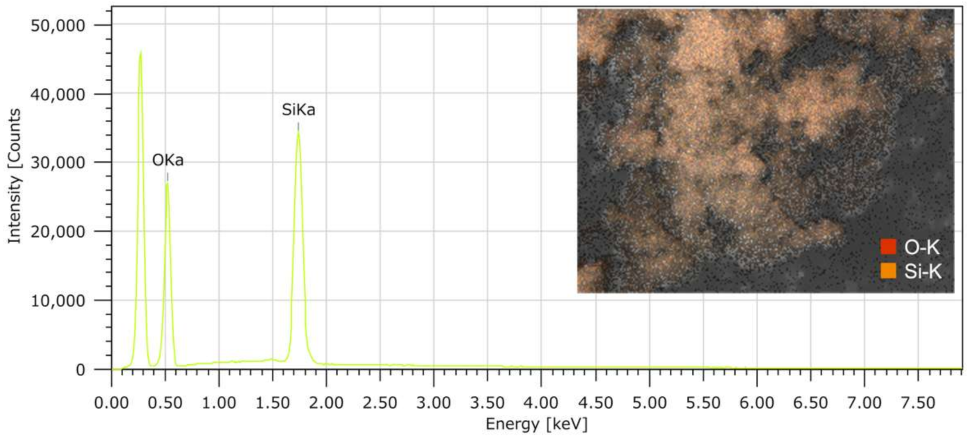

Figure S8. EDX spectrum and elemental composition of the SiO_2 nanoparticles.

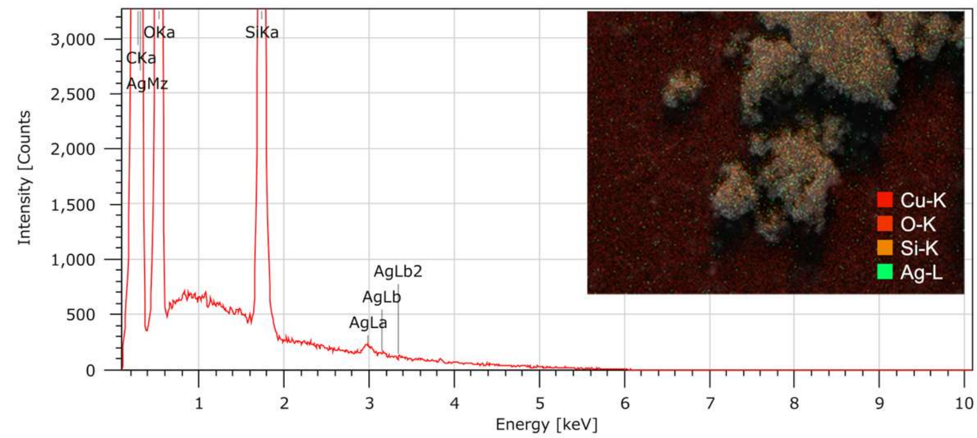

Figure S9. EDX spectrum and elemental composition of the SiO_2–Ag.

EDX analysis confirmed the presence of Ag on the SiO_2 nanoparticles. The atomic composition determined from the EDX analysis is summarised in Table S1.

Table S1. Atomic composition of SiO_2–Ag nanoparticles determined by EDX.

Element	Line	Atom%
C	K	54.63 ± 0.18
O	K	32.77 ± 0.19
Si	K	12.30 ± 0.11
Ag	L	0.30 ± 0.02

The Ag/Si atomic ratio obtained from the EDX analysis was used to estimate the number of Ag satellite nanoparticles per SiO_2 core, assuming spherical SiO_2 cores (206 nm), spherical Ag nanoparticles (5.6 nm), amorphous SiO_2 (2.2 g/cm^3), and bulk metallic Ag (fcc, 10.49 g/cm^3). Based on these assumptions, the number of Ag satellite nanoparticles was semi-quantitatively estimated to be approximately 460 per SiO_2 core.

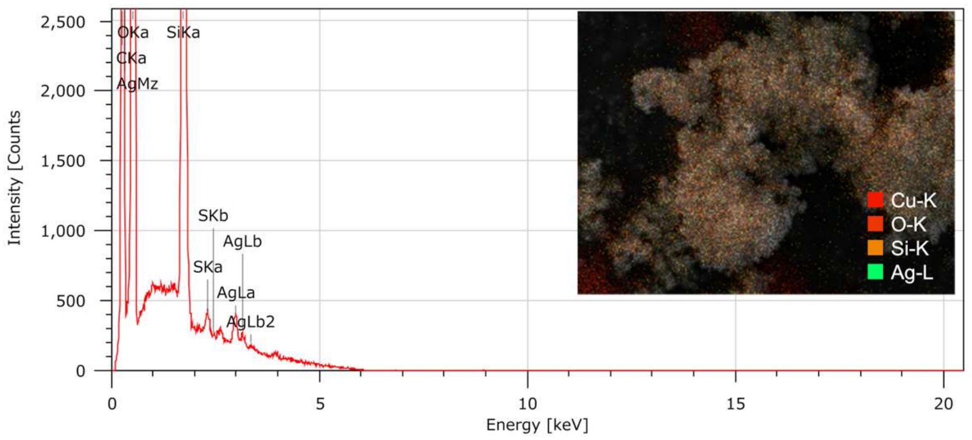

Figure S10. EDX spectrum and elemental composition of the SiO_2–Ag–RB.

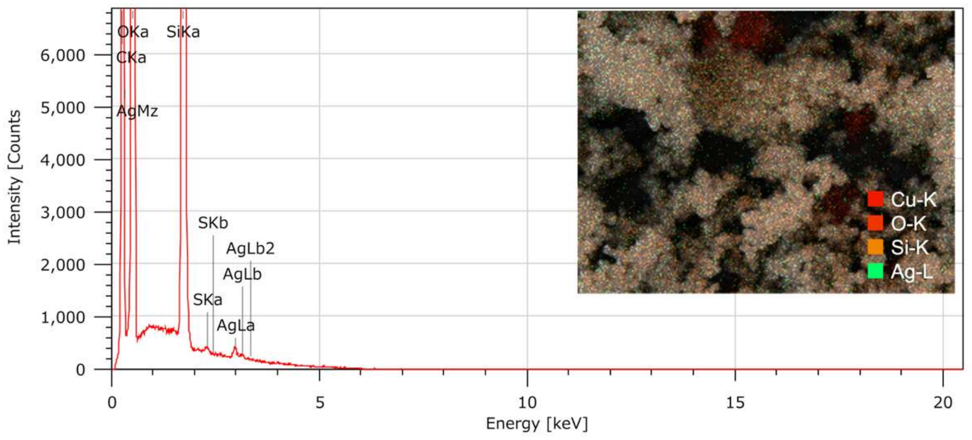

Figure S11. EDX spectrum and elemental composition of the SiO_2–Ag–PpIX.

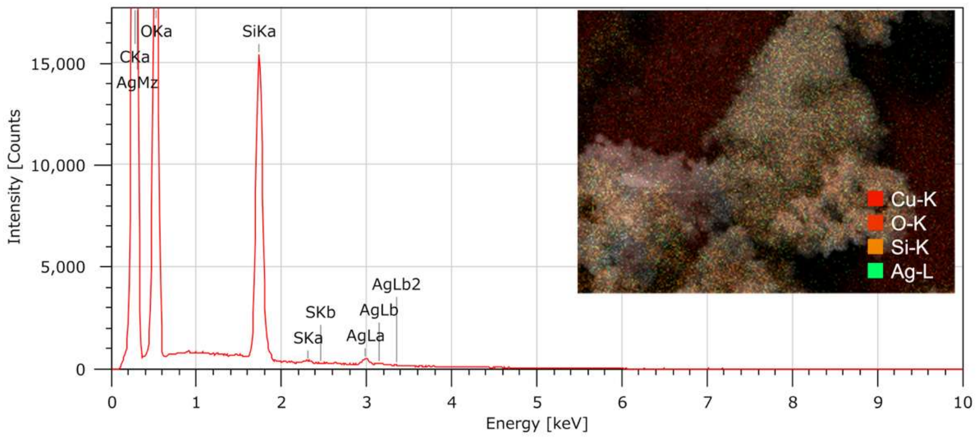

Figure S12. EDX spectrum and elemental composition of the SiO_2–Ag–C3CA.

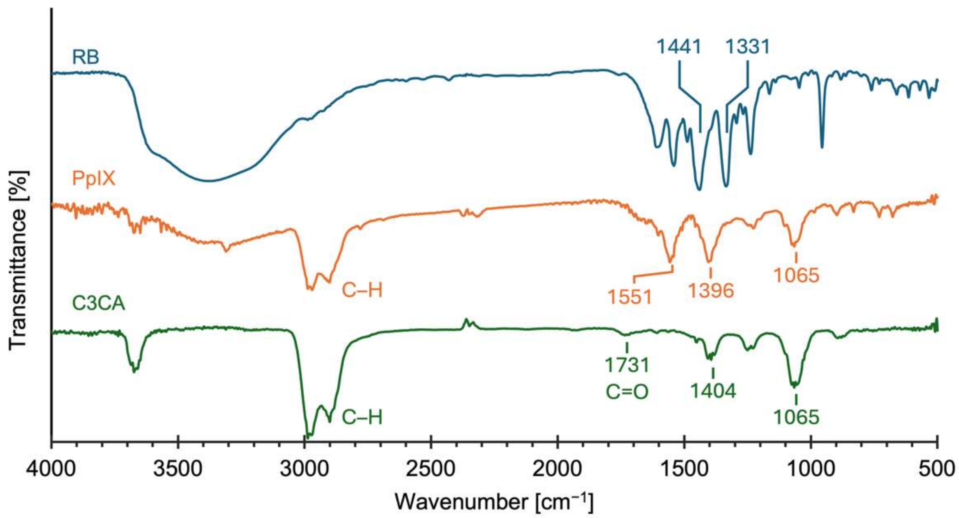

Figure S13. FTIR spectra of the free dyes. Spectra are vertically offset for clarity.

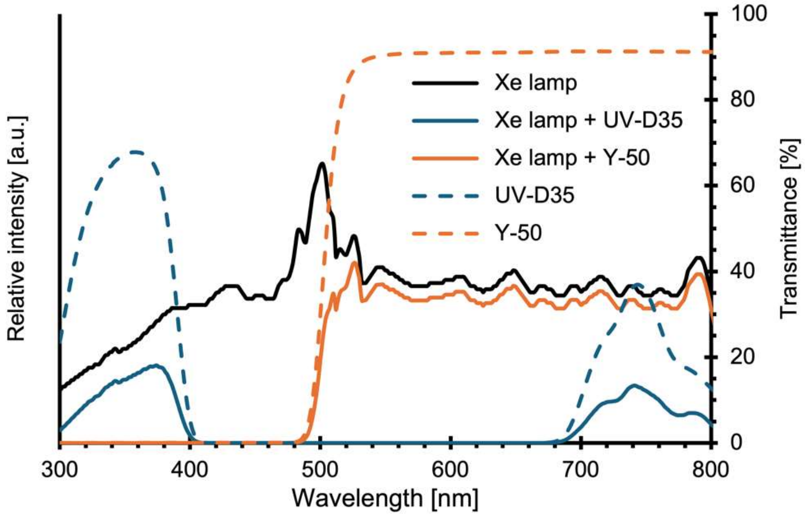

Figure S14. Spectral intensity distributions of the Xe lamp before and after passing through the UV-D35 and Y-50 optical filters (solid lines). The transmission spectra of the corresponding filters are shown as dashed lines.
